# Feasibility of Smartphone-Based Badminton Footwork Performance Assessment System

**DOI:** 10.3390/s20216035

**Published:** 2020-10-23

**Authors:** Ya-Lan Chiu, Chia-Liang Tsai, Wen-Hsu Sung, Yi-Ju Tsai

**Affiliations:** 1 Department of Physical Therapy, National Cheng Kung University, Tainan 701, Taiwan; yalanchiu0527@gmail.com; 2 Institution of Physical Education, Health and Leisure Studies, National Cheng Kung University, Tainan 701, Taiwan; andytsai@mail.ncku.edu.tw; 3 Department of Physical Therapy and Assistive Technology, National Yang-Ming University, Taipei 112, Taiwan; whsung@ym.edu.tw; 4 Institute of Allied Health Sciences, College of Medicine, National Cheng Kung University, Tainan 701, Taiwan

**Keywords:** smartphone, badminton, footwork, acceleration

## Abstract

Footwork is the most fundamental skill in badminton, involving the ability of acceleration or deceleration and changing directions on the court, which is related to accurate shots and better game performance. The footwork performance in-field is commonly assessed using the total finished time, but does not provide any information in each direction. With the higher usage of the smartphones, utilizing their built-in inertial sensors to assess footwork performance in-field might be possible by providing information about body acceleration in each direction. Therefore, the purpose of this study was to evaluate the feasibility of a smartphone-based measurement system on badminton six-point footwork. The body acceleration during the six-point footwork was recorded using a smartphone fixed at the belly button and a self-developed application in thirty badminton players. The mean and maximum of the acceleration resultant for each direction of the footwork were calculated. The participants were classified into either the faster or slower group based on the finished duration of footwork. Badminton players who finished the footwork faster demonstrated a greater mean and maximum acceleration compared to those who finished slower in most directions except for the frontcourt directions. The current study found that using a smartphone’s built-in accelerometer to evaluate badminton footwork is feasible.

## 1. Introduction

Badminton is a popular racket sport globally [1]. The performance characteristics of badminton include quick movements over short distances, high-intensity repeated movements, and agile foot movements to maximize shot accuracy [2]. During gameplay, athletes must move to specific points on the court and return to the center in the most efficient manner to ensure the quality of each shot [3,4]. Footwork agility has been proven to be related to gameplay performance [5]. Players who can run and change directions faster have enhanced game performance. Therefore, badminton footwork is always emphasized during gameplay and training. Footwork includes six directions on the court: moving to the right and left frontcourt, the right and left midcourt, and the right and left backcourt [6]. Different stepping and lunge strategies as well as arm movements may be used for different directions. The two frontcourts are relatively simple to reach, as moving forward is more intuitive than moving backward. Forehand and backhand stroking may be used, respectively, at the two sides (left and right) of the court. Thus, the ability to accomplish each direction of the 6-point footwork might be different for each player. Assessing the footwork performance in each direction becomes important.

An efficient evaluation of footwork performance is crucial in order to provide quantitative data on in-field performance. However, to date, methods for the assessment of footwork performance on the badminton court remain preliminary. Typically, only the total time to finish the six-direction footwork is obtained using a stopwatch, without collecting any more detailed information, such as the speed of movement in each direction [7]. Depth analyzing the performance of footwork in each direction would benefit the training of players and the work of coaches as movements on the badminton court are high frequency and dynamic.

Most relevant studies in laboratory settings have used optical motion analysis systems to investigate the swing motions involved in shots [8,9], but only a few studies have examined the biomechanics of the lunge motion. Kuntze et al. (2010) utilized an optical motion analysis system and force platform to investigate the kinematics and kinetics of the lunge task. Three badminton-specific lunge tasks, including kick, step in, and hop lunges, were compared in nine male players. The results indicated that the step-in lunge reduced the muscular demands of lunge recovery and the hop lunge produced greater positive power, representing a more efficient lunging method [4]. Lin et al. (2017) compared the joint loading during the right forward lunge motion of an underhand net lift between professional and amateur badminton athletes [10]. Their results revealed that professional players exhibited greater knee joint moments in the sagittal plane and frontal plane compared to amateur athletes [10]. To the best of our knowledge, no study has analyzed the lunge movement in different directions or overall footwork performance. Although motion capture systems can provide highly accurate and detailed information on human motions, these systems have some limitations. The devices are typically large, expensive, and limited to experimental environments, making them unsuitable for badminton courts [11]. Moreover, setting up motion capture systems (e.g., the calibration and fixation of reflective markers) is often complex and time-consuming.

To overcome these limitations, inertial measurement units (IMUs) are increasingly being utilized because of their smaller size, simple operation, and lack of location limitation [12]. The interference produced by the IMUs is much lower than that of the reflective markers of the motion capture system. IMUs may be more practical for use on the court [13,14]. Several studies have utilized IMUs to evaluate sports performance, including agility, speed, power, and sports techniques [12,13,14]. A recent review by Cammomilla et al. (2018) indicated that utilizing IMUs is a reliable method to measure sports performance in-field [12]. IMUs can be used for various purposes, including motor capacity assessment, technique analysis, and activity classification. IMUs have also been applied in various sports, such as running, swimming, skiing, tennis, and baseball. However, studies using IMUs to investigate badminton motions only focused on the swing motions during shots [13,14,15]. Jaitner and Gawin collected the acceleration data of the racket, forearm, and arm during smash movement in 26 professional badminton players [15]. The acceleration resultant for the corresponding smash movement was calculated, and the maximum and integral acceleration was used to represent the smash performance. The results revealed that the resultant racket acceleration was highly correlated with the shuttlecock velocity. Furthermore, the racket acceleration and negative acceleration of the forearm could be used to differentiate between international and national players.

Few studies have utilized IMUs for assessing agility performance or confirmed that IMUs can provide reliable and valid values to represent the shuttle run speed and ability to change directions [16,17,18]. Chika et al. (2017) employed the reactive agility task, which requires the participants to navigate a set of cones in response to a vocal cue. Inertial data were collected from body-worn inertial sensors placed on multiple locations, including the feet, shanks, thighs, sacrum, torso, forearms, biceps, and head. That study demonstrated that the kinematic data from body-worn IMUs can be used to quantify performance [18]. In 2015, McGinnis et al. analyzed agility performance using an accelerometer fixed to the sacrum. The results indicated that inertial sensors could provide quantified biomechanical metrics for speed and agility performance in agility assessment tasks [7]. In 2017, Zafriou also evaluated the agility performance of military staffs using feet-mounted inertial sensors.

However, some limitations remain when using conventional IMUs on badminton courts, including the power supply for the personal computer and sensor station, as well as the limited range of data transmission via Bluetooth. Although IMUs are considerably more affordable than motion analysis systems, their convenience and availability remain a concern for coaches and players [17]. As the popularity of smartphones surges, more and more researchers have utilized the high-quality built-in inertial sensors of smartphones to measure postural sway, joint range of motion, or gait spatiotemporal parameters [19,20,21]. Numerous studies have confirmed that smartphones’ built-in sensors are valid and reliable, and can be applied for measuring human motions [20,22]. Because of their convenience, smartphones might represent an innovative measurement tool for badminton footwork performance on the court.

Therefore, the primary purpose of this study was to develop and verify the feasibility of a smartphone-based badminton footwork performance measurement tool. The current findings could benefit both players and coaches during their daily training and follow up by providing them with quantitative information of each direction of the footwork performance, and a specific training program could be designed accordingly.

## 2. Materials and Methods

### 2.1. Participants

A total of 30 badminton players participated in the study, including 22 men and 8 women (Table 1). The average age was 20.7 (1.6) years old. The average height was 1.70 (0.07) m. The average weight was 62.1 (7.6) kg. The average body mass index was 21.3 (1.8). The average training time per week was 4.4 (2.5) h. The average experience of badminton was 6.63 (4.52) years. All the participants were recruited from the school team, department team, or badminton class from the university where the study was conducted. All the participants were required to be familiar with six-point footwork. Participants were excluded if they had any history of surgery or injury in the lower extremities in the preceding 6 weeks. To evaluate the discrimination ability of the assessment system, the participants were classified into two groups according to the median completion time of the footwork task: a fast and a slow group [18]. On the basis of the completion time of the six-point footwork, 15 players were classified into the fast group (12 men and 3 women), with the remaining 15 players classified into the slow group (10 men and 5 women). No significant differences were observed in their anthropometric data and average training duration per week (*p* > 0.05). Players in the fast group had a significantly shorter duration for completing the footwork than the slow group did (*p* < 0.001).

### 2.2. App Development

The application (app) was designed using the Android Studio 4 to acquire data from the accelerometer of the smartphone and to be operated by the users themselves. Because the Android system has a higher market share worldwide than other operating systems, we decided to develop the app under the Android operating system to maximize the future usability [23]. The self-developed app includes the following functions: introduction, measurement, and sensor testing. The introduction provides information on the purpose and procedures of the app, including instructions on how to attach the smartphone to the body. After selecting the measurement function, the user is provided with a 3-s count down as a preparation phase. A sound indicates when the user should start performing the designated footwork. Upon finishing the six-point footwork, the user must long click the finish button in the app; subsequently, the data are automatically saved to the smartphone’s built-in memory. The sensor testing function evaluates the sampling rate of the smartphone to ensure that the built-in inertial sensors are appropriate for the measurement.

### 2.3. Instrument

**Figure 1:**
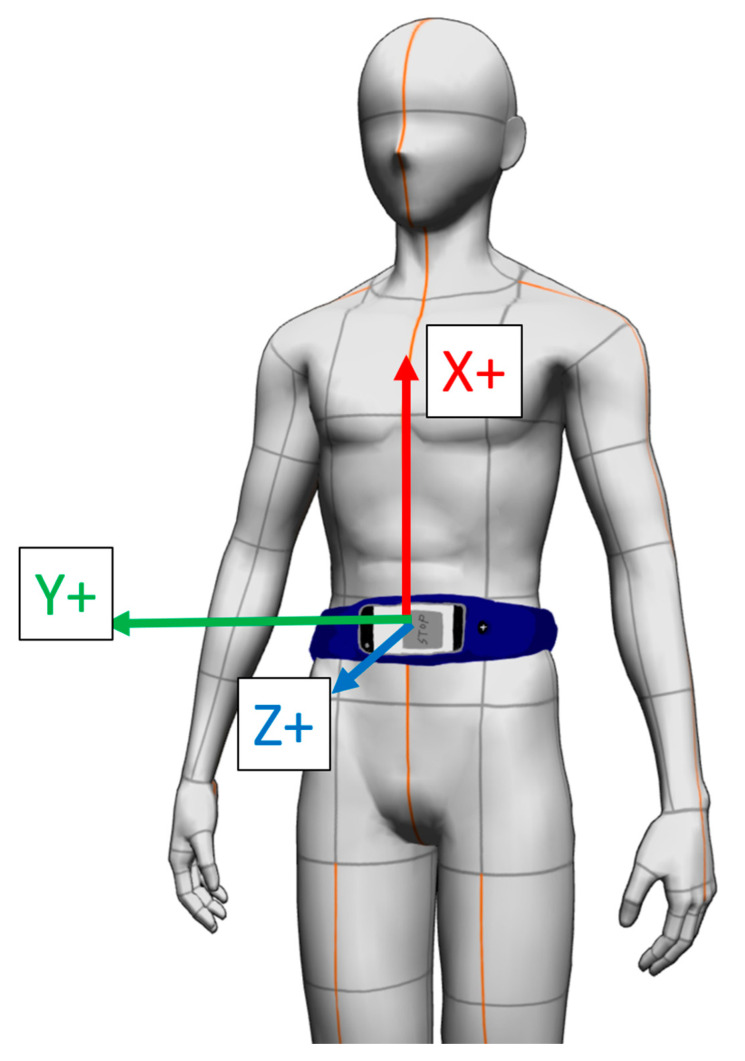
The orientation of the smartphone.

The ASUS Zenfone 6 was used in this study because of the steadiness of the built-in sensor sampling rate compared to other brands such as hTC in our pilot study. The built-in accelerometer was selected because related literature has proven the utility of accelerometers in evaluating sports-related performance [19,24]. The built-in accelerometer provides data on three axes—x, y, and z—with the highest sampling rate of up to 200 Hz. The placement of the smartphone and axial orientation of the built-in accelerometer in relation to the participants are presented in Figure 1.

### 2.4. Data Process

The acceleration data obtained from the accelerometer were processed using the MATLAB R2019a (The MathWorks Inc., Natick, MA, USA) software. Footwork performance was indicated as the mean and maximum of the acceleration resultants in each direction of the footwork task and during the whole task. The acceleration resultant was calculated, since the body was turning three-dimensionally while performing footwork [25,26,27]. The mean acceleration resultant represented the overall ability to accelerate or decelerate the body during the footwork task. The maximum acceleration result indicated the maximal capacity that an individual has to speed up or down at an instantaneous time point during the footwork task. The 3-s data, recorded when participants remained stationary before the start, were averaged as the offset of each axis. The acceleration resultant (*AR*) was calculated using the following formula:

$$AR=\frac{\sum_{i=1}^{n}\sqrt{{\left(x_{i}-x_{offset}\right)}^{2}+{\left(y_{i}-y_{offset}\right)}^{2}+{\left(z_{i}-z_{offset}\right)}^{2}}}{n}.$$

### 2.5. Procedures

**Figure 2:**
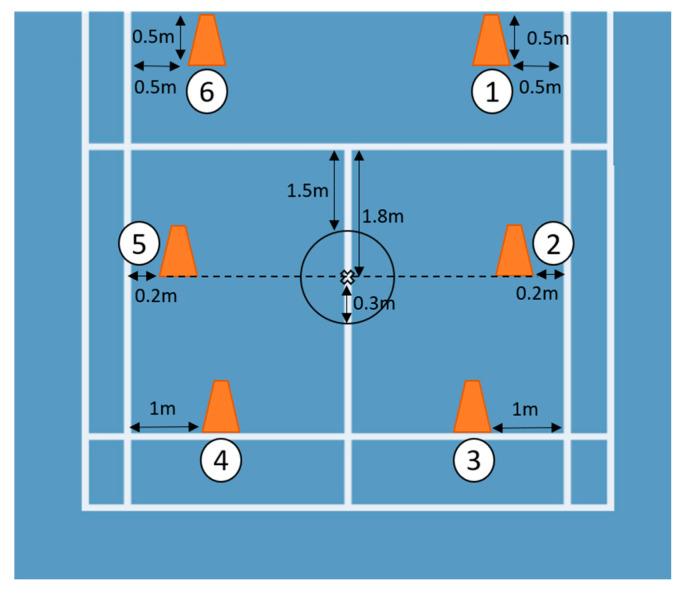
The footwork trial was designed followed by instruction from the school team coach. Six cones were placed in the corresponding locations.

This study was approved by the Institutional Review Board of National Cheng Kung University Hospital. Eligible participants were informed of the purpose and experimental procedures of the study and signed a consent form prior to participation. Data collection was conducted on a standard badminton court in the university. The functions of the app were first introduced to each participant. Participants were required to be able to operate the app independently. Subsequently, the smartphone was attached to the belly button (the standardized location) with a waistband (Figure 1). The footwork trial was designed followed by instructions from the school team coach. Six cones were placed in the corresponding target-locations (Figure 2). Participants were instructed to touch the four cones, two on the frontcourt and two on the midcourt, with their rackets. The backcourt section was considered complete when the participant’s foot stepped over the long serve line. The participants were instructed to finish the footwork in clockwise order, starting in the right frontcourt and finishing in the left frontcourt. A fixed order (non-random) of footwork was decided on in this study to prevent the cognitive or reactive involvement of the participants. After one or two practice trials to familiarize the participants with the app operation, each participant performed a total of three trials of six-point footwork using the smartphone. Sufficient resting intervals of one minute between trails were provided for each participant. The duration of each trial was recorded by the same investigator using a stopwatch, which is the most common method to assess footwork in-field.

### 2.6. Statistical Analysis

The normality assumption for parametric statistical analysis was tested using the Shapiro–Wilk test. Data are presented as means and standard deviations. Independent *t*-tests were used to compare the demographic differences between the fast and slow groups, as well as to determine whether the smartphone system could discriminate the differences in footwork performance between the two groups. In addition, the effect size (Cohen’s *d*) and 95% confidence interval of each variable of interest were reported. All the data were analyzed using SPSS (V.22.0, IBM Corp., Armonk, NY, USA). The level of significance was set a priori at α = 0.05.

## 3. Results

**Figure 3:**
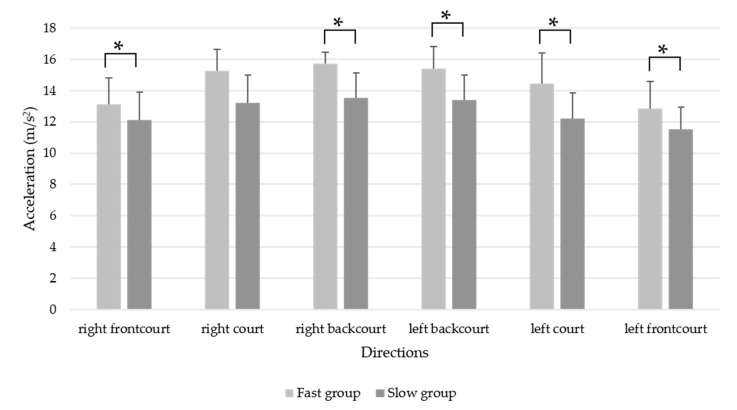
Mean acceleration (m/s^2^) for the whole footwork and in each direction between the fast and slow groups. * *p* < 0.05.

The results showed that utilizing the smartphone’s built-in accelerometer could differentiate the performance between the faster and slower groups based on the mean and maximum acceleration result. The means of the acceleration result for the entire footwork task and in each direction are presented in Table 2 and Figure 3. Except for the right frontcourt direction, significant group differences were observed for the entire task and in every direction. Players in the fast group had a significantly greater mean acceleration than those in the slow group did, and the effect sizes ranged from 0.75 to 1.70.

**Figure 4:**
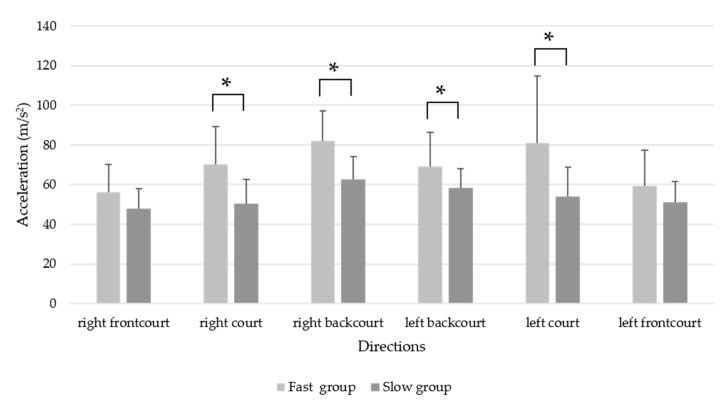
Maximum acceleration (m/s^2^) for the whole footwork and in each direction between the fast and slow groups. * *p* < 0.05.

The maximums of the acceleration result for the entire footwork task and in each direction are presented in Table 3 and Figure 4. Players in the fast group had significantly higher maximum accelerations than those in the slow group did for the entire footwork task and in most directions, with effect sizes ranging from 0.77 to 1.91. However, no differences were observed in the right and left frontcourt directions.

## 4. Discussion

The current study aimed to assess the feasibility of a straightforward footwork evaluation system utilizing a smartphone’s built-in sensor. To extend the usage to the general public, this system was specially designed for ease of use and with an embedded calculation function. Efficient performance of six-point footwork on the court is associated with competition results and is vital for badminton players. However, to date, no precise and convenient assessment method is available for assessing footwork on the court other than the total completion time of the task. In this study, we developed a smartphone-based assessment system that employs the built-in accelerometer and a self-developed app to quantify the on-court footwork performance in each direction. The current results revealed that the smartphone’s built-in accelerometer and the developed app could be used to differentiate the performance between the players who finished the six-point footwork faster and slower according to the parameters of the average and maximum acceleration achieved.

Researchers have employed in-field inertial sensors for measuring movements or performance in a wide range of sports, such as baseball, ice skating, running, and tennis. Spatiotemporal parameters, center of mass kinematics, segmental orientation and joint kinematics have been analyzed [12]. Acceleration data collected from the sacrum can provide the vertical velocity and position of the initial and final conditions during vertical jumping [16]. Moreover, the shoulder, elbow, and wrist trajectories during baseball pitching can be measured using an elbow-mounted inertial sensor [28]. An accelerometer attached to the trunk has also been considered to represent the whole-body center of mass. Most of the studies cited in the current paper have used variations in acceleration amplitude to measure performance [25,26,29]. Stetter et al. (2019) investigated the feasibility of body-worn accelerometers to discriminate skating players with different skill levels during a 30-m forward skating sprint. The accelerometer on the skate was used for defining skating phases and the accelerometer on the waist was used to calculate stride propulsion. Players with a higher skill level had larger stride propulsion compared to the recreational players, indicating a higher ability to generate an impulse to the body during skating [27].

To the best of our knowledge, no study has utilized acceleration to quantify the performance of badminton footwork. Only a few studies have used accelerometers to examine speed or agility during sports. In 2015, McGinnis et al. recruited 32 participants from the local university population to analyze the agility performance by using an accelerometer. The inertial sensor was fixed to the participants’ sacrum with a belt before a slalom run. The course was composed of seven cones spaced 5 m apart to define five changes of direction. The displacement, velocity, and acceleration of the participants were measured. The results indicated that inertial sensors could provide quantified biomechanical metrics for speed and agility performance in agility assessment tasks [7]. In 2017, Zaferiou used foot-mounted inertial sensors to collect tangential acceleration data for assessing the running agility. A group of recreational athletes were recruited to perform an outdoor obstacle course runs which included a five-cone agility drill with a series of five turns. The results revealed that the athletes with a shorter running duration had a larger tangential acceleration range. High performers made sharper turns and had larger changes in body speed and larger tangential acceleration ranges, enabling them to generate larger horizontal ground reactions during the turn phases [17]. These two studies measured the accelerations during agility drills with multiple changes of direction. The current findings were consistent with these two previous studies demonstrating that players with better performance had larger mean and maximum acceleration.

In this study, the players who finished the footwork faster demonstrated a larger mean and maximum acceleration result than did the players who finished slower in most directions, except for the right and left frontcourt. The results support that badminton players may have different performance among each direction of the footwork, and it is necessary to develop a method for assessing the footwork performance in each direction. Players who finished the footwork slower still had a similar acceleration ability to the players who finished faster in the forward directions. The possible explanation is that the right or left frontcourt directions, alongside the forehanded or backhanded swing, are maybe the most common and easily performed movements among all six directions for players. Forward stepping and lunging is a more intuitive direction for individuals to initiate acceleration. A relatively simple footwork strategy in the frontcourt directions may not challenge most players in this study. Therefore, no differences in the frontcourt directions between the two groups were found. The discrepancies between the two groups were observed in the sideward and backward directions, suggesting agility training for those who finished footwork slower could emphasize on these specific directions.

The findings of the current study highlight that using the built-in accelerometer could provide more information on footwork performance in each direction than only the completion time using a stopwatch. Badminton players may have different acceleration or deceleration ability depending on the footwork directions. The readily available smartphone and our self-developed app could easily provide the badminton players information of their acceleration or deceleration ability in each direction of footwork. Such a system assists the players and coaches to notice their direction of agility weakness on the court. A scientific-based and individualized training program may thereby be designed and implemented. The self-developed app system could also record the footwork performance of an individual day to day. The long-term changes of footwork performance could be followed which may assist the coaches to examine the effects of different training programs or help to monitor the health or injury conditions of the players. The self-developed app in the current study, which acquires the body acceleration data from the built-in accelerometer of a smartphone, enables a more comprehensive assessment of footwork performance in each direction of the court and may have considerable implications for sports in-field

## 5. Limitations

Several limitations in this study should be acknowledged. Only built-in accelerometer data were obtained; thus, only the linear aspect of performance was evaluated. Performance when changing directions or turning was not assessed in this study. Although our results showed that the acceleration resultant of the body could provide sufficient information on footwork in each direction, adding the angular data would definitely provide even more details of the agile performance. Future research could consider obtaining the gyroscope data from the smartphone to assess the angular performance of footwork.

The sequence of the footwork directions was not randomized in this study, and all participants were required to move from the right frontcourt in a clockwise direction to the left frontcourt. The clockwise design was to avoid the additional cognitive demand to the participants while memorizing the order of the directions or reacting to sudden instructions in different directions. The results might be less affected by the variability of the participants. However, a non-random order of footwork directions may induce bias such as fatigue or learning. The randomization of footwork directions is required in future studies. The overall finished duration of the footwork in this study was measured using a stopwatch, which may over- or underestimate the time and thereby influence the results. Future studies may consider using the light barriers to obtain more accurate and reliable time data for the footwork.

Finally, the badminton players recruited in this study were mainly from the university campus and were, therefore, relatively young. This lack of diversity in participants might limit the generalizability of the current findings. Future studies could consider recruiting players from diverse sources with various skill levels, including amateur and professional players.

## 6. Conclusions

The current study confirms that using a smartphone’s built-in accelerometer to evaluate badminton performance is feasible. The self-developed app system can acquire body acceleration data and provide more information regarding each direction of footwork. Badminton players who finished the six-point footwork faster demonstrated larger mean and maximum acceleration results than players who finished slower in most directions of the footwork task, except for the frontcourt directions. This system could be an easy to use alternative assessment system for the badminton court which might further benefit players and coaches when designing their training programs.

## Figures and Tables

**Table 1:** Descriptive data of participants. Mean ± standard deviation.

	Total (*n* = 30)	Fast Group (*n* = 15)	Slow Group (*n* = 15)	*p* Value
Sex (M:F)	22:8	12:3	10:5	0.409
Age (years)	20.7 ± 1.6	20.1 ± 1.6	21.3 ± 1.5	0.051
Height (m)	1.70 ± 0.07	1.73 ± 0.06	1.68 ± 0.08	0.086
Weight (kg)	62.1 ± 7.6	62.2 ± 7.2	62.0 ± 8.3	0.950
BMI (kg/m^2^)	21.3 ± 1.8	20.8 ± 2.1	21.8 ± 1.4	0.112
Training time (hours/week)	4.4 ± 2.5	5.3 ± 2.4	3.5 ± 2.5	0.062
Experience of badminton (years)	6.6 ± 4.5	6.7 ± 4.1	6.6 ± 5.0	0.937
Finished time (s)	14.8 ± 2.5	13.4 ± 2.43	16.3 ± 2.81	<0.001 *

BMI, body mass index; M, male; F, female. * *p* < 0.05.

**Table 2:** Mean acceleration (m/s^2^) for the entire footwork task and in each direction for the fast and slow groups. Mean ± standard deviation.

Footwork	Fast Group (*n* = 15)	Slow Group (*n* = 15)	*p* Value	Effect Size (Cohen’s *d*)	95%CI
Total	14.45 ± 1.00	12.69 ± 1.38	<0.001 *	1.48	0.86–2.66
Right frontcourt	13.13 ± 1.66	12.13 ± 1.78	0.124	0.57	−0.29–2.29
Right court	15.25 ± 1.41	13.20 ± 1.77	0.002 *	1.30	0.86–3.25
Right backcourt	15.71 ± 0.74	13.55 ± 1.60	<0.001 *	1.70	1.23–3.10
Left backcourt	15.39 ± 1.41	13.41 ± 1.59	0.001 *	1.33	0.85–3.10
Left court	14.43 ± 1.97	12.20 ± 1.67	0.002 *	1.19	0.87–3.60
Left frontcourt	12.83 ± 1.97	11.54 ± 1.41	0.035 *	0.75	0.10–2.48

* *p* < 0.05.

**Table 3:** Maximum acceleration (m/s^2^) for the entire footwork task and in each direction for the fast and slow groups. Mean ± standard deviation.

Footwork	Fast Group (*n* = 15)	Slow Group (*n* = 15)	*p* Value	Effect Size (Cohen’s *d*)	95%CI
Total	101.55 ± 19.10	72.32 ± 10.37	<0.001 *	1.91	17.57–40.87
Right frontcourt	56.33 ± 14.05	47.88 ± 9.94	0.068	0.69	−0.65–17.55
Right court	70.29 ± 19.08	50.57 ± 12.14	0.002 *	1.51	7.66–31.78
Right backcourt	82.20 ± 14.95	62.80 ± 11.32	<0.001 *	1.46	9.48–29.32
Left backcourt	69.10 ± 17.18	58.29 ± 9.72	0.043 *	0.77	0.37–21.25
Left court	81.03 ± 33.71	53.94 ± 14.82	0.010 *	1.04	7.21–46.98
Left frontcourt	59.47 ± 18.00	51.11 ± 10.61	0.132	0.56	−2.69–19.41

* *p* < 0.05.
